# Karyological evidence for diversification of Italian slow worm populations (Squamata, Anguidae)

**DOI:** 10.3897/CompCytogen.v7i3.5398

**Published:** 2013-09-23

**Authors:** Marcello Mezzasalma, Fabio Maria Guarino, Gennaro Aprea, Agnese Petraccioli, Angelica Crottini, Gaetano Odierna

**Affiliations:** 1 Dipartimento di Biologia, Università di Napoli Federico II, Via Cinthia I- 80126 Napoli, Italy; 2 CIBIO, Centro de Investigação em Biodiversidade e Recursos Genéticos, Campus Agrário de Vairão, R. Padre Armando Quintas, 4485-661 Vairão, Vila do Conde, Portugal

**Keywords:** Karyotype, chromosome banding, 16S rRNA, *Anguis*, Italian Peninsula

## Abstract

A karyological analysis on six Italian populations the slow worm (*Anguis veronensis* Pollini, 1818) was performed and their genetic differentiation at the mitochondrial 16S rRNA gene fragment from a Spanish sample has been assessed. The Italian populations were karyologically uniform, all showing 2n=44 elements, of which 20 were macrochromosomes and 24 microchromosomes. Comparison with literature data on Central European populations showed a difference on the morphology of the 10^th^ chromosome pair: submetacentric in Italian populations and telocentric in the Central European ones. Our analysis showed the presence of a fragile site on chromosomes of this pair, suggesting its propensity for structural rearrangements. Analysis of the 16S rRNA gene fragment showed uniformity among Italian populations (uncorrected genetic distance of 0.4%), and their genetic distinctness from the Spanish individual (uncorrected genetic distance of 4.2%). Our results confirm the existence of two different *Anguis fragilis* Linnaeus, 1758 lineages, each one characterized by a different cytotype.

## Introduction

Until recentlythere were only two recognized species of the genus *Anguis* Linnaeus, 1758 in the Palaeartic region: *Anguis cephallonica* Werner, 1894 and *Anguis fragilis* Linnaeus, 1758, commonly known as slow worms. The first species was considered a Mediterranean endemic restricted to the Peloponnese and some Ionian islands, while the second (*Anguis fragilis*) was considered a widespread taxon distributed from Western Europe to NW Iran and from the Mediterranean coast to Scandinavia, with a broad altitudinal distribution, ranging from the sea level up to 2300 m above sea level (Gasc et al. 1997, Sindaco et al. 2006). Two recent studies (Gvoždik et al. 2010, 2013) based on the analysis of mitochondrial and nuclear genes and morphology redefined the distribution and the phylogenetic relationships of various populations of the genus *Anguis* and identified five main distinct evolutionary lineages: *Anguis fragilis* sensu stricto (present in Austria, Bosnia and Herzegovina, Bulgaria, Croatia, Czech Republic, France, Germany, Great Britain, Greece, Hungary, Italy, Macedonia, Montenegro, Norway, Poland, Portugal, Serbia, Slovakia, Slovenia, Spain, Sweden and Switzerland), *Anguis graeca* Bedriaga, 1881 (in Albania, Greece and Montenegro), *Anguis cephallonica* (limited to the Peloponnese), and *Anguis colchica* (Nordmann, 1840) (widely distributed from Eastern Europe to Iran) and subdivided into three main lineages (*Anguis colchica colchica*, *Anguis colchica incerta* and *Anguis colchica orientalis*) each with a geographically distinct distribution; and more recently *Anguis veronensis* Pollini, 1818 (present in Italian peninsula and some areas from south-eastern France). Both the mitochondrial (mtDNA) and the nuclear (nuDNA) analyses analyses identified *Anguis cephallonica* as the sister lineage of a wider clade comprising *Anguis fragilis*, *Anguis colchica* and *Anguis graeca* (Gvoždik et al. 2010), while the position of the Italian lineage still remains unresolved (Gvoždik et al. 2013). The proposed species overall parapatric distributions, excluding some apparently isolated populations of *Anguis fragilis* sensu stricto from Greece (Gvoždik et al. 2010). In a previous karyological study (Gigantino et al. 2002) on the herpetofauna from the Matese Regional Park (Campania, Southern Italy) showed that local slow worm populations had a different chromosome formula when compared with available karyological data of *Anguis fragilis* (Dalq 1921, Margot 1946). Those karyological data, indeed scarce and dated, referred to referred to Central European populations of *Anguis fragilis* and described a karyotype of 2n=44 elements, of which 20 were macrochromosomes (the first, the fourth and the fifth pairs shaped as metacentric and the rest as telocentric elements) and 24 microchromosomes, arm number (A.N.)=48. The populations of the Matese Regional Park differed in showing as submetacentric the elements of the tenth macrochromosome pair (A.N.=50). On the basis of the recent finding of Gvoždik et al. (2013) and our previous karyological data (Gigantino et al. 2002), we extended the chromosome analysis to other Italian populations. The results of this comparative karyological study are here presented together with the re-worked analysis on populations from Matese Regional Park. In addition, since in Balkan Peninsula two endemic *Anguis* species were found (Gvoždik et al. 2013), we also perfomed a molecular analysis of a fragment of the mitochondrial 16S rRNA gene in order to test the genetic uniformity of the studied Italian specimens.

## Material and methods

Eleven individuals from six geographically distinct Italian localities were analysed in this work: Travacò Siccomario (Pavia, northern Italy), one male; Ancona (central Italy), two males; Valle Agricola (Caserta, southern Italy), one male and one female; Giffoni Valle Piana (Salerno, southern Italy), one male and one female; Ruvo del Monte (Potenza, southern Italy), two males and one females; Monte Cocuzzo (Cosenza, southern Italy), one males. As already successfully performed (Gigantino et al. 2002), adult individuals were sexed in the field by means of hemipenis extroversion before their release. For the samples of the Matese Regional Park we benefitted from stored chromosome suspensions obtained from organs (see Odierna et al. 2004 for details) kept in the herpetological collection of one of the co-author (G.F.M.). For all other samples, chromosomes were obtained by establishing blood cultures using a freshly collected blood sample (100–500 µl), drawn on the field from the caudal vein, incubated for 5 days in 3 ml of peripheral blood karyotyping medium (Biological Industries). Colcemid (100 ng/ml) was added to culture two hours before harvesting. After washing in Phosphate Buffer Saline (PBS) 1x, and incubation in hypotonic solution (KCl 0,075 M) per 30 min, cells were fixed in 3:1 methanol-acetic acid. This non-invasive method, useful for both karyologycal and molecular investigations, allows the avoidance of specimen sacrifice and animals were released at capture sites immediately after the sampling. In addition to standard staining method (5% Giemsa solution at pH 7) we performed various chromosome banding techniques: Ag-Nor banding (Howel and Black 1980), C-banding (Sumner 1972), C-banding+CMA_3_+DAPI staining (Odierna et al. 2004), Chromomycin A_3_-methyl green staining (Odierna et al. 2007); G banding performed on 10 days old slides by a brief incubation (10–30 sec) in a 0,05% trypsin solution (Odierna et al. 1994). For Late Replicating banding pattern (LR), bromedeoxyuridine (BrdU; 35 µg/ml) was added to blood cultures during the last six hours and differential staining was revealed by staining chromosomes with 4% Giemsa solution in 2% 4Na-EDTA for two minutes (Odierna et al. 2004).

**Figure 1.**
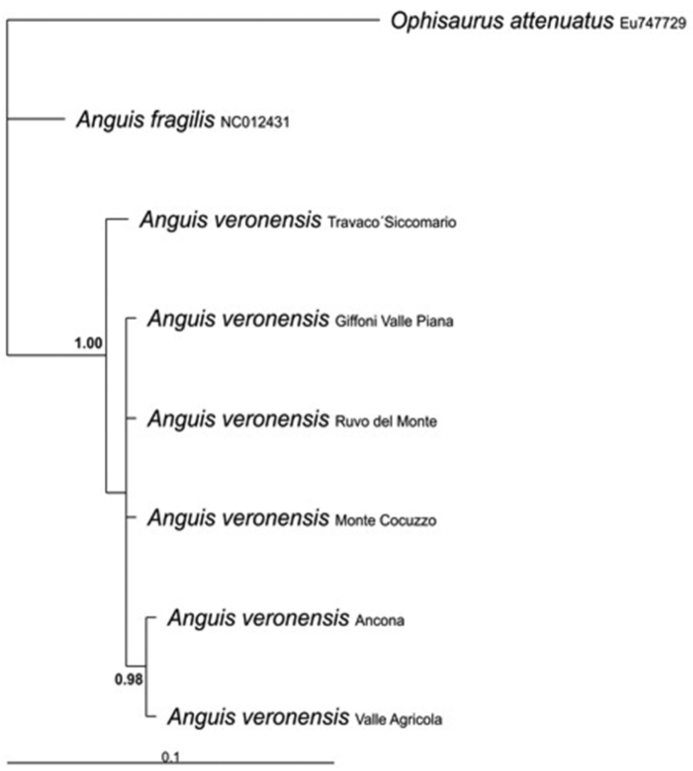
70%-majority consensus tree derived from a Bayesian inference analysis of 321 bp of the mitochondrial 16S rRNA gene. *Ophisaurus attenuatus* was used as outgroup. Sequences retrieved from GenBank are marked with their accession numbers.

Total genomic DNA was extracted from blood cells using conventional phenol-chloroform method (Sambrook et al. 1989). A fragment of ca. 320 bp of the mitochondrial 16S rRNA gene was amplified for one individual for each studied population using the primers 16Sa5’ - CGCCTGTTTACCAAAAACAT - 3’ and 16Sb 5’- CCGGTCTGAAACTCAGATCAGT- 3’ (Palumbi 1996). Amplification consisted of an initial denaturation step at 94°C for 5 min, followed by 36 cycles of denaturation at 94°C for 30 s, annealing at 55°C for 30 s, and elongation at 72°C for 30 s, followed by a final extension of 72°C for 7 min. Amplicons were sequenced on an automated sequencer ABI 377 (Applied Biosystems). Sequences were blasted in GenBank and chromatograms were checked by eye and edited, when necessary, using Chromas Lite© and the BioEdit sequence alignment editor (version 7.0.5.3; Hall 1999). Newly provided sequences were compared with a homologous sequence of a Spanish individual from Vilarmiel (Lugo province, Galicia, Spain) available in GenBank (Albert et al. 2009; NC012431). A homologous sequence of *Ophisaurus attenuatus* Baird, 1880 (EU747729) from Castoe et al. (2008) was add to the alignment and used as outgroup in the phylogenetic analysis. The alignment of all sequences required the inclusion of gaps to account for indels in only a few cases. All newly determined sequences were submitted to the European Nucleotide Archive (ENA) (accession numbers: HG003678-HG003683). Uncorrected pairwise distances (*p*-distances transformed into percent) within individuals of *Anguis veronensis* and between species were computed using MEGA, version 5.05 (Tamura et al. 2011). Bayesian analyses were performed in MrBayes 3.1.2 (Ronquist and Huelsenbeck 2003). The HKY model was determined by AIC in jModeltest (Posada 2008) as the best-fitting model of substitution. We performed two runs of 10 million generations (started on random trees) and four incrementally heated Markov chains (using default heating values), sampling the Markov chains at intervals of 1,000 generations. Stabilization and convergence of likelihood values was checked by visualizing the log likelihoods associated with the posterior distribution of trees in the program Tracer (Rambaut and Drummond 2007). The first three millions of generations were discarded and seven thousand trees were retained post burn-in and summed to generate the majority rule consensus tree (Fig. 1).

## Results and discussion

**Figure 2.**
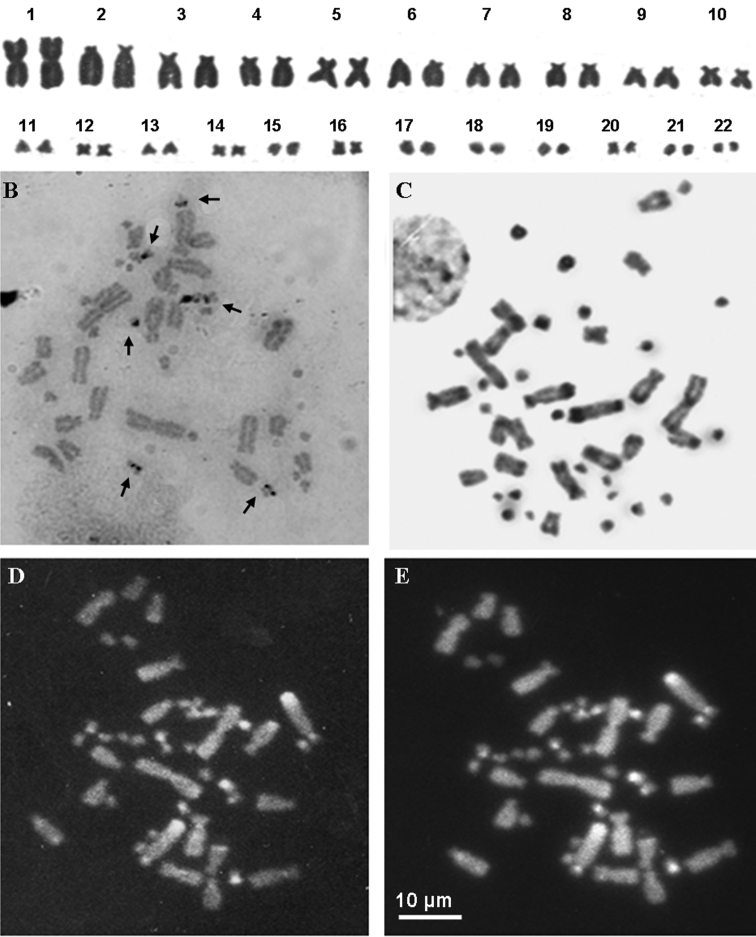
**A** Giemsa stained karyotype of an individual from Valle Agricola **B** Ag-NOR-banding stained metaphase plate of an individual from Ruvo del Monte **C**, **D**, **E** metaphase plate of an individual from Valle Agricola stained with C-banding (**C**) and C-Banding+ CMA_3_ (**D**)+ DAPI (**E**). Arrows in (**B**) point at NOR loci.

**Figure 3.**
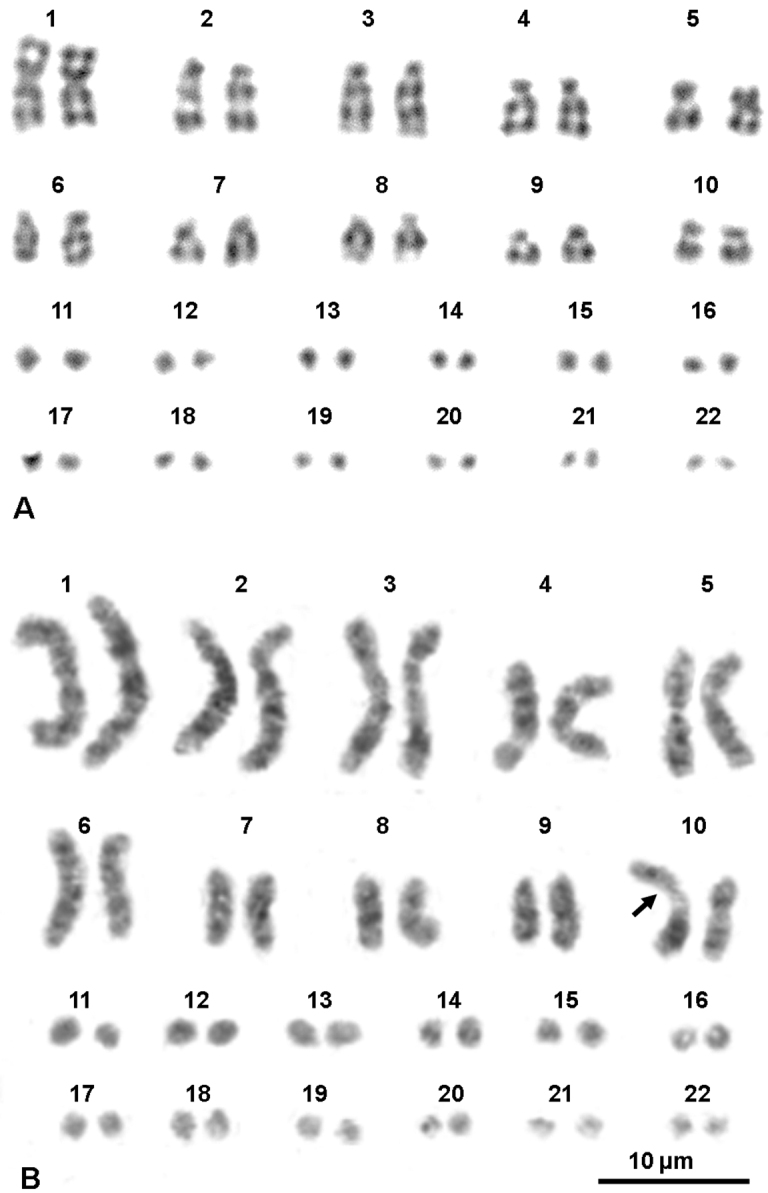
Karyotypes of a male from Travacò Siccomario (**A**) and of female from Ruvo del Monte (**B**) stained with G-banding and replication pattern, respectively. The arrow in (**B**) points at a fragile site.

The alignment of the analysed 16S rRNA gene fragments showed a minimum of 14 nucleotidic substitutions (11 transitions and 3 transversions) and 2 insertion/deletions between samples from Italy and Spain, corresponding to an average uncorrected genetic distance of 4.2%, thus confirming the genetic distinctiveness of the Italian populations. On the contrary, the analyzed Italian specimens were genetically very uniform and showed an intraspecific uncorrected divergence of 0.4%, whereas their uncorrected genetic distance from the outgroup, *Ophisaurus attenuatus*, was 12.8%. According to Gvoždik et al. (2010) the average genetic divergence at the ND2 mitochondrial locus among the several *Anguis* species is about 7%, while the maximum intraspecific distance within the various *Anguis colchica* clades is 4.4%. Unfortunately, we did not use the same molecular marker, but the observed 4.2% distance at the more conserved 16S rRNA gene fragment among Spanish and Italian samples provide further evidenced for the differentiation of these two lineages. Even if the phylogenetic analyses resulted in a tree (Fig. 1) with largely unresolved relationships, we could recover a good support for the monophyly of the Italian specimens, as well as for a clear mithocondrial segregation between them and the extra-Italian *Anguis* samples analysed in this study. Metaphase plates suitable for chromosome analysis were obtained from four out the six investigated populations (Travacò Siccomario, Valle Agricola, Ruvo del Monte and Monte Cocuzzo). All individuals showed a karyotype of 2n = 44 elements, comprising ten pairs of macrochromosomes and twelve pairs of microchromosomes. The first macrochromosomes pair was metacentric, the fourth and fifth were submetacentric and the others were telocentric with the exception of the tenth pair (Fig. 2A; Table 1). Chromosomes morphology did not differ between sexes and in any of the various Italian localities studied. The Ag-NOR banding revealed the presence of NOR loci on three microchromosome pairs in all examined specimens (Fig. 2B, arrows). This condition is considered an apomorphic character in Squamata, derived from a plesiomorphic single NOR locus (Porter et al. 1994, Aprea et al. 1996). The C-banding + Giemsa and the C-banding+fluorochromes colorations rrevealed small pericentromeric C-bands on almost all macrochromosomes and on three pairs of microchromosomes, namely those NOR bearing ones (Fig. 2C, D, E). Both G and LR-banding did not reveal any difference among analysed populations or between sexes (Fig. 3A, B). However, LR banding highlighted the presence of a uncondensed trait in three out of 20 examined metaphase plates (15% on the short arms of one chromosome of the tenth pair). This uncondensed trait was observed only on metaphase plates from cultures with BrdU addition and probably is a fragile site. In fact, it is known that BrdU promotes the expression of fragile sites (e.g. Sutherland 1979, Stone and Stephens 1993) (Fig. 3B). Fragile sites are usually due to a DNA strand breakage and represent hot spots for recombination (Glover and Stein 1988). The presence of a fragile site on the tenth chromosome suggests the propensity for this chromosome for structural rearrangements. By chance, this chromosome pair karyologically differentiates Italian slow worm populations from Centro-European ones, this pair being shaped as submetacentric in the former populations and as telocentric in the Centro-European ones. A pericentric inversion can account for the different morphology of the tenth chromosome pair. Due to the absence of karyological data on other congeneric species, the polarity of chromosome rearrangements cannot be unambiguously assessed. The role of chromosome rearrangements in speciation is heavily debated (White 1978, Odierna et al. 1996, Simard et al. 2009), as it is difficult to ascertain if chromosome changes occur after species diversification or if they are involved in the speciation process in itself, acting as proximate cause of diversification. Chromosome polymorphism as well as interspecific differences characterized by a single chromosome inversion are known in lizards (Odierna et al. 2004, Kupriyanova et al. 2008, Aprea et al. 1996). Thus, the difference in the chromosome 10^th^ pair might have occurred during or after the diversification between Italian and central European lineages. It should be also taken into consideration that the different morphology of the 10^th^ pair may be a consequence of a centromere repositioning, so far observed only among different species using comparative genome analysis (Montefalcone et al. 1999, Cardone et al. 2007).

**Table 1.** Chromosome relative length (RL), centromeric index (CI) and chromosome shape (CS) of studied samples of *Anguis veronensis*. The values of RL and CI are expressed as mean ± standard deviation. Chromosome morphology was measured according to Levan et al. (1964). For the microchromosomes 11–22 only their complessive RL value is provided.

**chrom.**	**RL**	**CI**	**CS**
**1**	15,2 ± 3,5	0,45 ± 3,6	metacentric
**2**	11,7 ± 2,7	0,10 ± 3,8	telocentric
**3**	9,7 ± 3,3	0,08 ± 4,4	telocentric
**4**	7,6 ± 3,1	0,07 ± 3,5	telocentric
**5**	7,4 ± 3,4	0,42 ± 3,6	metacentric
**6**	7,1 ± 3,1	0,07 ± 4,1	telocentric
**7**	6,2 ± 3,1	0,10 ± 3,1	telocentric
**8**	5,7 ± 2,8	0,08 ± 2,9	telocentric
**9**	5,1 ± 2,7	0,07 ± 4,0	telocentric
**10**	4,6 ± 3,2	0,34 ± 3,8	submetacentric
**11–22**	19,7 ± 6,8	–	–

To conclude, our karyological study is consistent and provides further support for the recently discovered molecular and morphological differentiation of the Italian slow worm lineage (Gvoždik et al. 2013), who ascribe the Italian populations to the recently resurrected *Anguis veronensis*.
